# High-frequency micro-viscoelasticity of biofluids by using optical trapping interferometry

**DOI:** 10.1007/s12551-026-01426-x

**Published:** 2026-03-09

**Authors:** Pablo Domínguez-García

**Affiliations:** https://ror.org/02msb5n36 grid.10702.34 0000 0001 2308 8920 Dep. Física Interdisciplinar, Universidad Nacional de Educación a Distancia (UNED), Madrid, 28040 Spain

**Keywords:** Biopolymers, Microrheology, High-frequency, Optical tweezers, Laser interferometry

## Abstract

Viscoelasticity is an essential characteristic of biomaterials like cells or tissues. The measurement of mechanical properties in biofluids requires experimental techniques using nanoscale local probe analysis and biological samples in small quantities. Optical trapping interferometry (OTI) tracks the Brownian motion of an optically trapped spherical microsphere, which explores the surrounding fluid driven by thermal energy. This technique provides a bandwidth in the microsecond range, which allows accessing the high-frequency regime in one-particle microrheology. In this paper, we review how the OTI equipment can be used to measure the micro-viscoelasticity of biofluids in the regime of high frequencies, in order to extract information about the dynamics of the individual biopolymers composing the material. We detail the implementation of this setup and its most recent applications in biophysics, alongside the development of the theory of Brownian motion and microrheology regarding the stochastic motion of the microprobe and the extraction of the mechanical properties of the fluid in which the particle is immersed.

## Introduction

For many years, scientists have been very interested in the mechanical characteristics of biological substances, like networks of proteins, tissues, and even individual living cells, and how they are connected to their internal, molecular mechanisms (Bao and Suresh 2003). An example of these efforts is the study of the cytoskeleton, which is composed by a dynamic network of polymers (Banerjee et al. 2020) and that provides shape and strength to the cells (Pritchard et al. 2014). This dynamic network changes as cells develop and mature, and plays an essential role in various cellular activities (Mizuno et al. 2009), whose mechanical alterations are related to illnesses, notably cancer (Mierke 2014).

However, studying these biopolymeric structures presents significant obstacles because of their diversity in size, structure, and mechanochemical activity (Burla et al. 2019). Their intricate shapes and sensibility to external conditions make it difficult to use standard methods to measure their structure and micromechanics. The development of innovative experimental techniques in the last decades (Furst et al. 2017) has allowed to overcome these challenges and limitations, and, therefore, the accessing to individual mechanical behavior of biopolymers.

All complex fluids and biomaterials exhibit rheological properties, meaning they have the capacity to deform in response to applied mechanical forces (Larson 1999). Materials classified as purely viscous fluids fully dissipate kinetic energy via viscous flow, characteristic of liquid-like behavior. Conversely, purely elastic materials store all input energy within entirely reversible deformations, similar to solid-like responses. A broad spectrum of soft materials exists between these two archetypes. These materials display both viscous and elastic attributes, hence their designation as viscoelastic. The manifestation of these viscoelastic properties depends on the temporal scale and the magnitude of the imposed stress (Ferry 1980). As the macroscopic viscoelastic behavior of materials arises from the microscopic responses of their constituent molecular components to external forces, rheology serves as an invaluable methodology for elucidating the microscopic dynamics inherent to living materials (Verdier 2003).

When investigating the rheology of complex fluids, especially when dealing with biofluids and living materials, the manipulation, positioning, and tracking of particles or objects of micro-nanometer scale sizes inside the fluid are often used. This field of research is called microrheology (Mason and Weitz 1995; MacKintosh and Schmidt 1999) and analyzes the Brownian motion (Einstein 1905, 1906) of these probe particles inside the fluid as an applied mechanical stimulus, leading to the estimation of its rheological properties in the microscale (Squires and Mason 2010). Microrheology has several advantages in comparison with bulk rheology: very small volumes of fluids can be used (on the order of $\mu$l), the measurements are made in the biological scale (nm-$\mu$m), and a wide spectrum of frequencies is available, including the range of high-frequencies ($\sim \,$MHz). The mechanical properties of complex fluids at such high frequencies may contain relevant information about living cells (Fabry et al. 2001), in biomaterials (Oelschlaeger et al. 2013), or in industrial applications (Vadillo et al. 2010).

A variety of microrheological techniques are at the disposal of the experimentalist (Waigh 2005; Gardel et al. 2005; Waigh 2016) but, in this review, we focus on Optical Trapping Interferometry (OTI). This set-up is basically composed by a laser interferometry equipment combined with optical tweezers, and allows to measure the motion of the probe with high spatial accuracy (nanometers) on a very short-time scale (microseconds), i.e., can be used for exploring microrheology in the high-frequency regime. Through this experimental set-up, Brownian motion in the range of the microseconds was investigated during the second half of the 2000s (Lukić et al. 2005, 2007; Jeney et al. 2008). Remarkably, it was later used for the experimental detection of the transition from ballistic to diffusive motion (Huang et al. 2011), and the first measurement of the resonances appearing due to hydrodynamic memory in Brownian motion (Franosch et al. 2011). The capabilities and limitations of this experimental equipment for the calculation of microrheological magnitudes in non-Newtonian fluids have been also determined (Domínguez-García et al. 2014, 2016; Butykai et al. 2017), allowing its application to study the micro-mechanical properties of biofluids (Domínguez-García et al. 2020, 2023, 2024).

In this review, we will describe how high-frequency microrheology has been used to study the micromechanics of some biomaterials and the dynamics of individual biopolymers in water suspensions. We first detail the advances in the knowledge of the Brownian motion at the microsecond scale, where the inertia and hydrodynamic effects cannot be neglected. After that, we will describe the OTI equipment and how it can be used for high-frequency microrheology. Finally, we will describe how this experimental set-up has been employed to investigate the microviscoelasticity of biofluids.

**Fig. 1:**
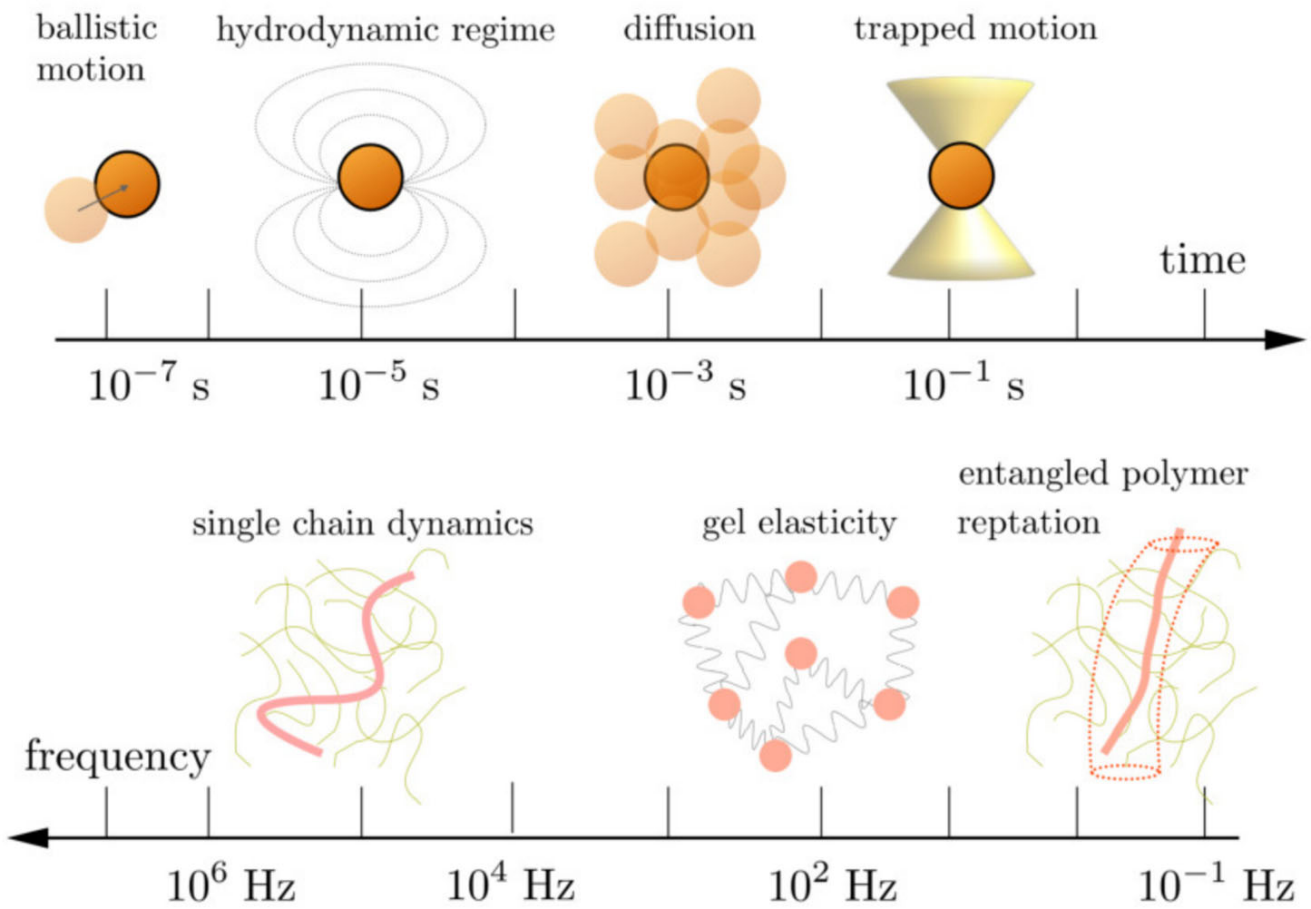
Top) Approximate characteristic time values for an optically trapped Brownian probe in a Newtonian fluid: at short timescales, the particle undergoes ballistic motion following the influence of the hydrodynamic effects; at intermediate times, the standard Brownian diffusion is observed, which is confined at higher timescales by the harmonic potential of the trap (Franosch et al. 2011). Bottom) In the frequency space, the observed mechanical behavior of a polymeric non-newtonian fluid also depends on the characteristic frequencies: at lower values, it is defined by the reptation of polymers in the tube model, following gel elasticity, and finally, at higher frequencies, by the single chain dynamics of polymers (Krajina et al. 2017)

### Brownian motion beyond Einstein

In 1828, Robert Brown wrote the following (Brown 1828): *“While examining the form of these particles immersed in water, I observed many of them very evidently in motion [..]. These motions were such as to satisfy me, after frequently repeated observation, that they arose neither from currents in the fluid, nor from its gradual evaporation, but belonged to the particle itself.”* Albert Einstein explained the Brownian motion in 1905 (and Smoluchowski in 1906 Smoluchowski 1906), without previous knowledge of Brown’s work, by developing a model where the motion of a small-sized particle floating in a fluid fluctuates because of the momentum transfer from the thermally excited molecules of the surrounding medium (Einstein 1905, 1906) This theoretical explanation leads to the diffusion coefficient, *D*, for the colloidal particle through the well-known Stokes-Einstein-Sutherland expression (Sutherland 1905), which is $D = k_B T/\gamma _0$, where $k_BT$ is the Boltzmann constant, *T* is the temperature, $\gamma _0=6\pi \eta a$ is the Stokes’ drag (Stokes 1851), $\eta$ is the fluid viscosity, and *a* the particle radius. In 1908, Langevin added a stochastic force term in a reformulated Newton’s force balance equation to model the random impacts of the fluid’s molecules in the colloidal particles (Langevin 1908). Perrin and his students confirmed these theoretical advances in 1908–1911 by experimentally determining the value of the Avogadro’s number (Perrin 1910) and, thus, the validation of molecular-kinetic theory (Hänggi and Marchesoni 2016).

However, Einstein already realized that if we watch a Brownian particle under the microscope closely enough, his description does not work, and the particle moves “ballistically” at short times, a behavior that can be derived from the Langevin equation by taking into account the inertia of the particle (Langevin 1908). Later on, it was discovered that the correlations between friction and velocity are non-instantaneous, the particle positions are correlated up to longer times (Vladimirsky and Terletzky 1945), the hydrodynamic vortices generated by the particle motion generate memory effects, and the particle velocity decays much more slowly than exponentially (Alder and Wainwright 1967), as should be expected for instantaneous friction. After all that, theoretical advances have been made when reconsidering the influence of the fluid’s mechanics to the motion of the Brownian particle (Hinch 1975; Pomeau and Résibois 1975; Clercx and Schram 1992). Experiments using dynamic light scattering in colloidal suspensions confirmed that the diffusion of colloidal particles is influenced by fluid mechanics, hence it is time-dependent (Boon and Bouiller 1976; Weitz et al. 1989; Kao et al. 1993).

Some additional time was needed to experimentally confirm that Brownian motion is quite different when observed at short timescales (see Fig. 1). At time scales nearby the microsecond, the influence of inertial effects and hydrodynamic memory is observable (Li and Raizen 2013). The full transition from the diffusive to the ballistic region was first experimentally determined in 2011 (Pusey 2011; Huang et al. 2011), and the short-time effects lead to the observation of “the color of Brownian motion,” which consists in a colored frequency-dependent component in the spectrum of the thermal forces (Felderhof 2009).

Therefore, Brownian motion has been a subject of intense research since Einstein’s breakthrough, and whose knowledge has been developed following different stages, being the current one the stage of application, i.e., the modern field of Soft Matter (Haw 2002). At this stage, the idea of using a Brownian particle as a probe for the determination of the properties of its local environment is established.

**Fig. 2:**
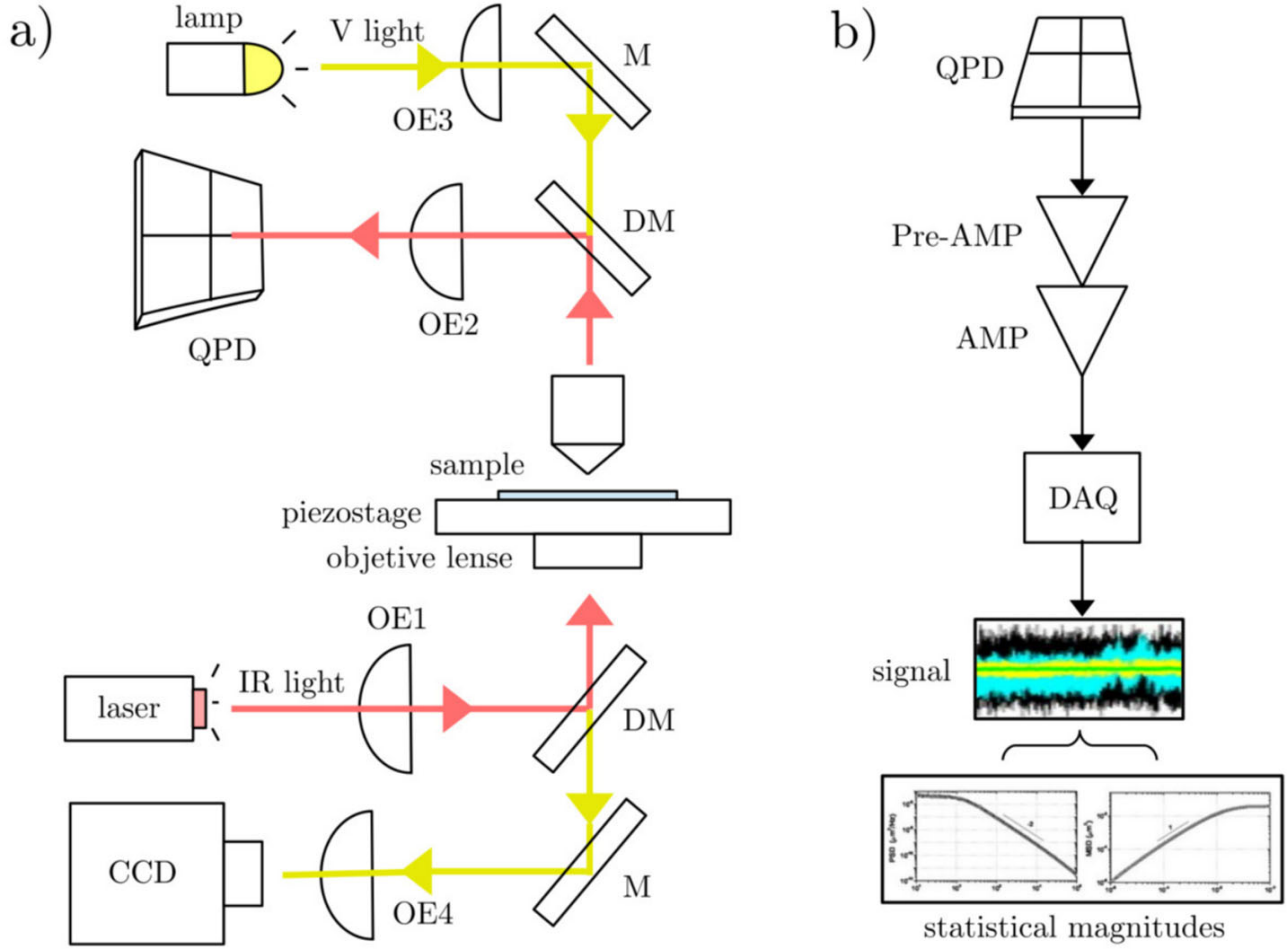
Example of an optical trapping interferometry setup (Guzmán 2008). **a** The arrows indicate the paths of the IR light from a Nd:YAG laser ($\lambda = 1064$ nm) and the visible light from a lamp. The optical elements (OE) are the following: OE1 contains neutral density filter and a beam expander, OE2 is formed by lenses, and neutral density filter in front of the quadrant photodiode (QPD), OE3 contains a lens and a diffuser, and OE4 is a tube lens. The elements marked with an M are lower mirrors, and DM are dichroic mirrors. **b** The intensity fluctuations of the probe are recorded in the QPD, converted to volts (Pre-AMP), amplified (AMP), and digitalized using an acquisition card (DAQ), returning a signal of position versus time which allows to calculate different statistical magnitudes related to the probe motion

## Optical trapping interferometry

During the decade of the eighties of the past century, two fundamental techniques for the experimental study of the atomic structure of molecules, especially in the context of biological structures, were developed: scanning tunneling microscopy (STM) and atomic force microscopy (ATM). These techniques can be considered under the category of scanning probe microscopy (SPM) because both use an object to scan the sample surface (Florin et al. 1997). However, a basic need for these apparatus to perform is to control the movement of the probe through some kind of mechanical device.

During the same decade, to answer to that need, Ashkin proposed the optical tweezers technique (Ashkin 1980), receiving the Nobel Prize in Physics in 2018 thanks to their subsequential and numerous applications in biotechnology, nanotechnology, colloid physics, or complex fluid rheology (Pesce et al. 2020). This technique allows the manipulation of small particles by optical trapping (Gennerich 2022), which is basically achieved by a focused laser beam incident on the probe. The light is dispersed when it interacts with the object and, since light carries an associated momentum and the total momentum of the system must be conserved, the particle readjusts its position, being trapped by the incident beam (Grier 2003). Since the applied optical potential is harmonic in the central region of the potential well (Svoboda and Block 1994; Richardson et al. 2008), it generates a very simple and useful Hookean external force in the center of the beam, i.e., $F(t) = -kx(t)$, where *k* is a constant to be determined, and *x*(*t*) is the temporal displacement of the probe. However, this restoring force has the potential to modify the motion dynamics of the Brownian particle (Lukić et al. 2007). On the contrary, thanks to this applied force, the effects in the motion dynamics caused by confining (Faucheux and Libchaber 1994; Jeney et al. 2008) can be avoided if the particle is moved far enough from the borders of the containing cell. In any case, the instrumental aspects and principles of optical trapping have been extensively reported in the literature (Svoboda and Block 1994; Sheetz 1998). The optical tweezers technique, when applied in aqueous solutions and dielectric probes (Ashkin et al. 1986; Ashkin and Dziedzic 1987), has been of great importance to study the response of colloidal particles in complex fluids, so that it has been possible to measure, for example, the viscoelastic properties of biopolymers (such as DNA) or the cell membrane (Verdier 2003).

By using optical tweezers, the photonic force microscopy (PFM) was developed as a new type of SPM, specifically designed to work with biological samples (Florin et al. 1997). This particular experimental technique is sometimes called optical trapping interferometry (OTI), since the optical tweezers are combined with a high-resolution interferometric position detection to record the trajectory of the Brownian probe embedded in a complex fluid. In OTI, the optical trap ensures that the particle remains within the detector range, but it has also the function of providing a light source for the position detection. This technique has been a fundamental experimental set-up for the experimental observations of Brownian motion in the short-time scale (Jeney et al. 2010; Grimm et al. 2012). The basic components of such an apparatus are an inverted light microscope with a 3D sample positioning stage, an infrared laser for the optical trapping, and a quadrant photodiode (QPD) for the measurement of the particle position.

As shown in Fig. 2, which describes a typical OTI configuration !‘(see details in the caption), the light path is divided in two: one for infrared trapping and detection, and a second for visible illumination and imaging light. A near-IR wavelength for the laser avoids the sample becomes excessively heated in the laser focus (Svoboda and Block 1994) and a CCD camera can also added for the control of the trapped particle.

The fluid to be studied, containing the microspheres, is located in a sample chamber mounted in the 3D piezo-stage. Different types of polymeric particles can be used for these kind of experiments (Li et al. 2024), usually composed by polystyrene, melamine resin, or silica with sizes in the rank from 100 nm to 10 $\mu$m (Furst et al. 2017). The particles can be suspended in Newtonian liquids, such as high-purity acetone or water, or viscoelastic fluids composed of polymers in water. For good trapping efficiency, a good difference is necessary between the refractive indices of the liquid and the material composing the probe (Franosch et al. 2011). In this kind of setup, only the motion of one particle is detected and analyzed, so a very small volume of the particle suspension has to be added to the fluid to maximize the average distance between particles and minimize their mutual influence (Lukić et al. 2005). When an isolated particle is trapped inside the liquid, the scattering from the light of the laser is measured using the QPD and converted into volts. After that, these intensity fluctuations are amplificated to span the dynamic range of the acquisition card. The position of the particle is measured by the interference pattern generated by the scattered and unscattered light detected in the QPD. By this methodology, it is possible to measure the motion of the trapped object in three dimensions (Gittes and Schmidt 1998; Pralle et al. 1999; Rohrbach and Stelzer 2002).

Afterwards, the motion of the Brownian particle is usually analyzed through statistical quantities, such as the velocity autocorrelation function (VAF), the power spectral density (PSD), the mean square displacement (MSD), and the position autocorrelation function (PAF) (Domínguez-García et al. 2013).

One of the essential points when using interferometry for one trapped particle is the implementation of the calibration process of the signal, i.e., the unit conversion for the position of the trapped particles from volts into meters. Calibration can be made through theoretical curves in a Newtonian fluid because the analytical solution of Langevin equations (Langevin 1908) depends only on a set of characteristic times, in the case of a spherical particle under an external harmonic potential with no-slip boundaries and hydrodynamic effects (Clercx and Schram 1992). Therefore, obtaining the volts-to-meter conversion factor is straightforward when the bead is immersed in a Newtonian fluid (Grimm et al. 2012; Butykai et al. 2015, 2017). A classic calibration methodology involves the calculation of the optical trap stiffness through the MSD characteristic plateau at long-times, the PAF (Domínguez-García et al. 2013), or by Lorentzian form of the PSD (Addas et al. 2004). If the constant *k* has been previously estimated, comparing both values permits to deduce the conversion factor. For complex fluids, an analytical solution of trapped particle equations of movement is not available because a more complex liquid than a Newtonian fluid depends on unknown parameters related to the medium viscosity and the friction memory kernel (Grebenkov 2011). However, this issue can be avoided if the mechanical behavior of the fluid is simple enough, like a Kevin-Voigt fluid (Domínguez-García et al. 2020), or by using the method of the double flow chamber (Bertseva et al. 2012; Domínguez-García et al. 2014; Domínguez-García and Jeney 2025). For some fluids, and when using an intense optical force, the plateau in the MSD can be assumed to mainly appear because of the external force, where the contribution of the elastic part of the fluid is negligible. Therefore, it is possible to calibrate all the curves (Domínguez-García et al. 2024) by applying that, in generic viscoelastic fluids, the MSDs under different trap strengths collapse and tend to the same value at very short-time values (Domínguez-García et al. 2014). Alternative methodologies can be employed for the calibration of similar types of set-ups (Florin et al. 1998; Capitanio et al. 2002; Fischer and Berg-Sørensen 2007; Atakhorrami et al. 2008; Dutov and Schieber 2013). Therefore, once the experimental approach is defined and controlled, it is assumed that any deviation from the expected Brownian motion of the particle can be interpreted as a response to the material properties of its complex environment (Gittes et al. 1997; Frey and Kroy 2005).

## High-frequency microrheology with OTI

By understanding the stochastic dynamics of spherical micro- and nanobeads immersed in a complex fluid, we can extract the mechanical properties of that medium. This field of investigation is called microrheology (Mason and Weitz 1995; Waigh 2005, 2016), as an extension in the microscale of classical rheology (Larson 1999; Han 2007; Mewis and Wagner 2012), and has been used in the last three decades in biophysics and soft matter (Mason et al. 1997; Fabry et al. 2001; Mezzenga et al. 2005; Squires and Mason 2010). Microrheology appears at the end of the nineties of the past century, and various experimental techniques quickly emerged to probe the material response in the micrometer-length scale using microliter sample volumes (Schnurr et al. 1997; MacKintosh and Schmidt 1999), remarkably video-microscopy (Crocker et al. 2000; Gardel et al. 2003), and laser scattering (Schnurr et al. 1997; Mason et al. 1997; Pine et al. 1988; Addas et al. 2004).

Most microrheology methods use tracer particles to locally deform the fluid sample for studying the response of the material by analyzing the trajectory of the probes. When microrheology is called active, the experimental technique uses an external driving force, like optical or magnetic tweezers (Bausch et al. 1999), to measure the response of the material through the control of the particle. However, in active materials, internal processes may affect the motion of the probe and, consequently, the determination of their rheological properties (Córdoba 2018), and several criticism has been made to the microrheology of living materials with active probes (Tassieri 2015).

On the contrary, passive measurements only use the probe’s thermal energy (Mizuno et al. 2008; Wilson et al. 2009), in the order of $k_B T$, to extract the mechanical properties. This methodology has the limitation that the surrounding medium has to be soft enough to be analyzed with only $k_B T$ of energy, but the advantage is a noninvasive methodology with minimal influence of the probe on the material. This is the situation in OTI, since the optical trapping only locates the particle in a certain region, and it is soft enough to minimally affect the motion of the probe at medium and short-time scales.

OTI only provides the trajectory of one particle, resulting in one-particle (1P) microrheology. The error values for the MSD from the probe’s measured trajectory are usually calculated by the blocking methodology (Flyvbjerg and Petersen 1989; Córdoba and Schieber 2022). Opposed to bulk rheology (Gardel et al. 2003), the measurements of 1P microrheology depend on the characteristic length scales of the system (Atakhorrami et al. 2014), and, therefore, the results obtained from macro and microrheology do not need to match (Buchanan et al. 2005b). To avoid these differences, two-particle (2P) microrheology (Levine and Lubensky 2000; Crocker et al. 2000) generates excellent results thanks to its detection of the fluctuations at large length scales. In any case, 1P microrheology has the added value of being able to isolate the mechanical properties of the fluid in the micro-scale of the tracer bead, i.e., in the local biological scale (Chen et al. 2003).

It is worth to briefly summarize how the mechanical properties of a fluid are obtained from the movement of the tracer particles, since the limitations of the measurements are intimately bounded to the applied theory. Here, classical rheological concepts are applied, where an oscillatory strain of frequency $\omega$ generates the measurable mechanical properties of the fluid through the elastic (storage) $G'(\omega )$, and the viscous (loss) modulus $G''(\omega )$ (Squires and Mason 2010), and where the complex modulus $G^*(\omega )=G'(\omega )+iG''(\omega )$ is related to the complex viscosity $\eta ^*(\omega )$ by $G^*(\omega )=i\omega \eta ^*(\omega )$. The theoretical ground to obtain the complex moduli is the generalizated Stokes-Einstein relation (GSER) (Mason and Weitz 1995), which connects the MSD of the probe particle with the complex modulus by using a generalized Langevin equation. The GSER in three dimensions is $G^*(\omega ) = k_B T/6 \pi a D^*(\omega )$, where *a* is the probe particle radius and $D^*(\omega )$ is the Fourier transform of the temporal-depending diffusion coefficient. Consequently, Furst et al. (2017) the practical expression $G^*(\omega ) = k_B T/ \pi a i\omega \, \overline{\text {MSD}}$ is derived, where $\overline{\text {MSD}}$ is the one-side Fourier transform of the MSD. For calculating the Fourier transforms from a limited range of data, the Mason’s approximation is normally used (Mason 2000), which assumes that the MSD follows a power-law behavior locally. Then, the mechanical properties can be calculated directly from the MSD of the particle. The GSER methodology, along with Mason’s approximation, has become standard in the field of microrheology, but theoretically equivalent alternatives for calculating the complex modulus exist in the literature (Evans et al. 2009). Their particular use and application depend on the type of measured data and its response to noise or error propagation. A classical approach is using the Kramers-Kronig relationships (Kronig 1926; Kramers 1927), which can be estimated through their generalization for viscoelastic fluids (Booij and Thoone 1982), or through the PSDs (Schnurr et al. 1997; Addas et al. 2004) by using efficient Filon methods for integrating oscillatory integrals (Shampine 2013; Domínguez-García et al. 2020).

However, the GSER has a number of limitations that have to be accounted for. The fundamental ones are the physical assumptions for its derivation. Firstly, the material has to be in thermal equilibrium, or close to it. The non-equilibrium behavior can be checked by using the probability distribution of the tracer displacements in function of the time lag, i.e., the van Hove correlation function. Most biological materials generate non-equilibrium dynamics through their internal processes, and, in such cases, the van Hove profiles will show variations from the Gaussian behavior (Mizuno et al. 2007; Stuhrmann et al. 2012). Secondly, the medium has to be considered as a continuum by the probe particle (Furst et al. 2017). This last assumption is essential for the applicability of the Stokes equation, and it is verified if the radius of the particle is greater than the characteristic length size of the material. Thirdly, the GSER neglects the coupling of the probe to the system by compressional modes (Addas et al. 2004). This issue, which has been addressed in the context of two-particle microrheology (Córdoba et al. 2013). Additionally, the derivation of the simplest expression for the GSER does not include external forces, such as the spring force exerted by optical tweezers. If the linear or nonlinear optical traps are not directly subtracted (Córdoba et al. 2012), the external optical trap will be observed as a component of the elastic modulus, $G'$, at low-frequencies, which may affect the fluid itself (Domínguez-García et al. 2016). Regarding the behavior of the elastic modulus, in high-frequency microrheology, a characteristic breakup in the elastic modulus at very high frequencies ($\omega >10^4$ Hz) is sometimes observed (Piechocka et al. 2010). The elastic component is very sensitive to artifacts (Savin and Doyle 2007; Domínguez-García and Rubio 2013), when it abruptly changes its curvature (Mason 2000), and it behaves poorly at very high frequencies because of the numerical calculations at low values of *t* (Mason 2000; Addas et al. 2004). The main advantage of OTI as an experimental technique, beyond the study of Brownian motion, is that it allows accessing the behavior of the material at the high-frequency range in the biological scale. The high-frequency regime allows to study the characteristic dynamics of the individual polymers composing the fluid (Krajina et al. 2017) through the power-law behavior of the complex moduli at such frequencies (Dasgupta et al. 2002; Buchanan et al. 2005a). In this frequency range, the loss modulus dominates the elastic one almost by two orders of magnitude, and the thermal fluctuations dominate over athermal ones. Consequently, regarding the viability and limitations of microrheology, the van Hove distributions should be Gaussian (Mizuno et al. 2007; Toyota et al. 2011), and the loss modulus shows a more consistent and precise power-law behavior than the elastic one.

However, the effects of inertial and hydrodynamic memory in the Brownian motion of the probe are not negligible in the microsecond scale (Franosch et al. 2011; Li and Raizen 2013). These high-frequency effects (Liverpool and MacKintosh 2005; Atakhorrami et al. 2008) have to be taken into account when obtaining the complex moduli, especially in terms of calculating a precise power-law exponent. An expression for the calculation of the complex modulus including these effects is available (Indei et al. 2012b, a) and it has been directly compared to the simplest GSER formula in the analysis of power-law behavior in fluids composed of semiflexible polymers (Domínguez-García et al. 2014), and for the study of the influence of the solvent in polymer dynamics in semidilute water suspensions (Domínguez-García and Jeney 2025).

### Biopolymers in aqueous suspensions

Therefore, the viscoelastic behavior in the high-frequency regime of an aqueous suspension of biopolymers reveals information about the molecular components of the biostructures in the fluid. Additionally to noninvasive techniques such as OTI or Diffusing Wave Scattering (DWS) (Maret and Wolf 1987; Pine et al. 1988; Gardel et al. 2003), other active experimental approaches have been carried out for studying the viscoelastic response of biomaterials in this regime of frequencies, f.e., living cells by a high-speed-AFM (Rigato et al. 2017), or the cytoskeleton by the force exerted by optical tweezers (Head et al. 2014; Staunton et al. 2019). By the application of an analogous technique to OTI, high-frequency microrheology of solutions of fd viruses revealed that their individual dynamics correspond to the one of semiflexible filaments (Addas et al. 2004), following a power-law behavior $G^*\sim \omega ^{3/4}$ at high-frequencies (Gittes and MacKintosh 1998). Most filamentous biopolymers are considered semiflexible, providing exponents very near to 3/4, e.g., when studying the motion of endogenous tracers inside cancerous and non-cancerous cells with OTI (Bertseva et al. 2012).

A classic example of a semiflexible polymer is actin, in particular, filamentous actin (F-actin), which is the most abundant protein in the cytoskeleton of eukaryotic cells and determinant for their mechanical properties (Bershitsky et al. 1997; Bao and Suresh 2003). The fluids composed by these biopolymers have been widely studied in the context of microrheology and Soft Matter during the last decades (Gisler and Weitz 1999; Crocker et al. 2000; Schmidt et al. 2000; Gardel et al. 2003; Koenderink et al. 2006; He and Tang 2011). F-actin solutions provide an example on how microrheology may return different results, in comparison with bulk rheology, depending on the characterization of the involved length scales (Buchanan et al. 2005b). In fact, an exponent value of 7/8 instead of the expected 3/4 for semiflexible structures has been observed using microrheology in these kind of fluids (Chae and Furst 2005; Koenderink et al. 2006; Atakhorrami et al. 2008; Tassieri et al. 2008, 2012; Grebenkov et al. 2013; Atakhorrami et al. 2014). The origin of this exponent value and the differences with classical rheology rely on the characteristic lengths of the network in relation to the size of the tracer particle. The 7/8 exponent is characteristic of local longitudinal fluctuations of the polymers, which can be considered as a measurement of the micro-mechanics of the fluid at the local biological scale (Chen et al. 2003).

**Fig. 3:**
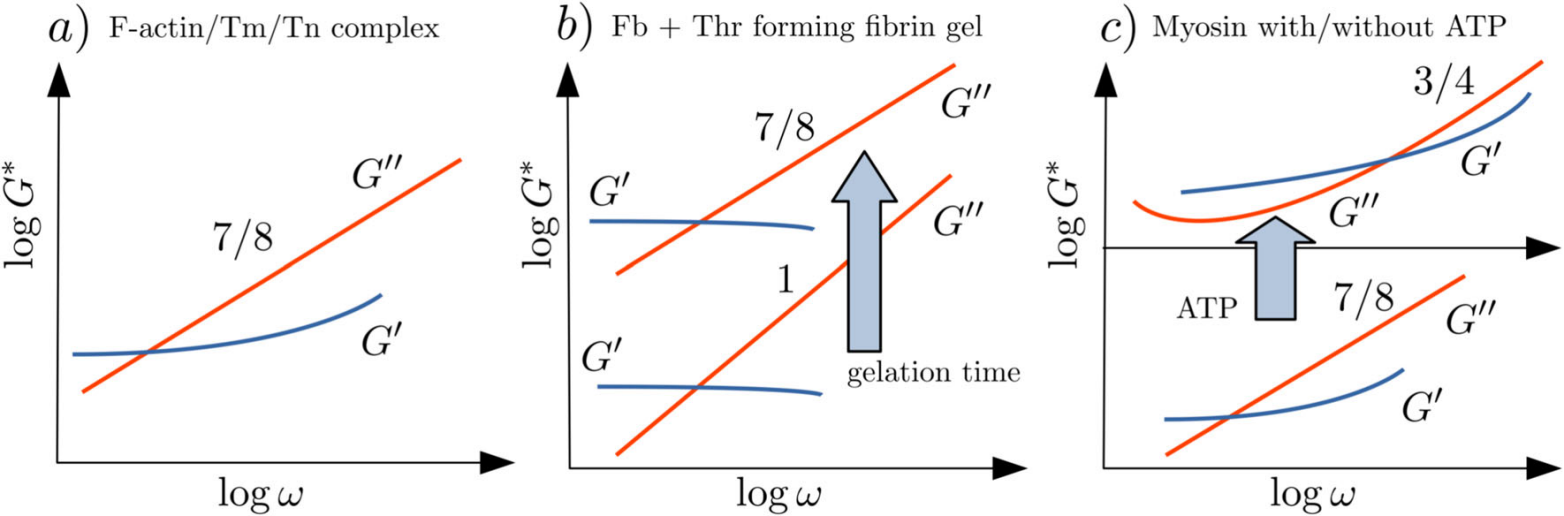
Qualitative curves representing the observed micromechanical behavior of aqueous solutions of F-actin, fibrin, and myosin using OTI (Domínguez-García et al. 2023, 2020, 2024). **a** Thin filament composed by the complex F-actin/Tm/Tn (see text), **b** an aqueous solution of fibrinogen with enzyme thrombin generating a fibrin gel, and **c** myosin solutions changing their micromechanics by adding adenosine triphosphate (ATP)

A similar viscoelastic fluid is a water solution of thin filaments, which are structures composed by tropomyosin (Tm) and troponin (Tn) coupled to F-actin in the presence of Ca$^{2+}$. This Tm/Tn complex has a role in muscle contraction by the interaction between myosin and actin (Ebashi et al. 1971), and stabilizes and increases the stiffness of the filament (Götter et al. 1996). The thin filament has a high-frequency micro-viscoelasticity very similar to single F-actin solutions (Domínguez-García et al. 2023), showing power-law values $\alpha$ between 0.75 and 0.9, which are compatible in average with the exponent 7/8, but with a marked dependence on several factors, such the interaction between the applied optical force and the depletion zone of the probe particles. These exponent values are less dispersed for the Tm/Tn complex, confirming some kind of mechanical stabilization in comparison to F-actin alone.

The exponent value 7/8 is also observed using OTI in fibrin networks at high gelation times (Domínguez-García et al. 2020). The fibrin gels are one of the main components of blood clots, and their mechanical behavior is important for understanding blood coagulation and the healing of open wounds. Fibrin aggregates are related to risk of bleeding, thrombosis, and even to cancer (Boccaccio 2006; Weisel 2004). For generating fibrin gels, the protein fibrinogen (Fb) is needed, which is polymerized by the enzyme thrombin (Thr). Physiologically, Fb is contained in the plasma, and Thr is activated when a wound appears in the body. By the action of this enzyme, Fb molecules generate fibrin monomers and then develop a three-dimensional fibrin gel (Kollman et al. 2009; Lim et al. 2008). In OTI experiments, the probe is introduced into an aqueous suspension of Fb, which is activated using Thr. The fibrin gel is formed around the probe, allowing to observe its stepwise formation: monomers, protofibrils, fibers, and network. The high-frequency microrheology shows that the fibrin gel behaves according to a simple Kelvin-Voigt type mechanical model at the first temporal steps of the polymerization, deviating to a 7/8 exponent at the final steps of the gelation process (Domínguez-García et al. 2020).

By modifying the characteristic length scales in the fluid, it is possible to observe the change of the exponent values from 7/8 to 3/4. In myosin aqueous solutions, this behavior appears when measuring the micro-viscoelasticity in the presence of sufficient Mg$^{2+}$ and in the absence and presence of adenosine triphosphate (ATP) (Domínguez-García et al. 2024). Myosin is a protein involved in the dynamical processes of eukaryotic cells (Kovács and Málnási-Csizmadia 2013) and drives muscle contraction by binding to F-actin (Korn and Hammer 1988). As a molecular motor, myosin uses the hydrolysis of ATP, which is the main source of energy in cells, in order to move the actin filaments and producing contractions in the actomyosin networks (Ebashi et al. 1971). Without the influence of ATP, myosin is a primary gelling protein used in the food industry (Yang et al. 2007), and its relaxed state may be connected to cardioprotective mechanisms (Hooijman et al. 2011). When experiments of myosin solutions are performed using OTI, a power-law behavior in the loss modulus with exponents 7/8 is obtained. However, this same experimental procedure, when adding 10 mM ATP generates a reconfiguration of the micro-viscoelasticity of the suspensions, generating a fluid behavior very similar to classical semiflexible polymers, like worm-like micelles in solution, with a 3/4 exponent at high frequencies (Buchanan et al. 2005a; Domínguez-García et al. 2014). Moreover, the complex moduli show that the inverse of the relaxation time is three times higher when using myosin from rabbit skeletal muscle than for pig cardiac muscle, which is a result compatible with a qualitative interpretation of the different mechanical behavior of cardiac and skeletal muscle. Figure 3 plots the approximate mechanical behavior of these viscoelastic biofluids. Thanks to the high-frequency regime reached by OTI, the changes in the single biopolymer dynamics composing the fluids are detected, reflecting the influence of different factors, such as gelation time or energy transduction.

## Conclusions and outlook

In summary, this review briefly discussed how an experimental technique originally developed to explore the limits of Brownian motion has been used in the last years for the study of the micro-mechanical properties of biofluids. Optical trapping interferometry (OTI) allows accessing high-frequency microrheology, revealing the dynamics of the individual polymers, which provide the viscoelasticity to the solution. Because of the extensive knowledge needed for the application of OTI as a useful microrheological tool, we review the fundamental aspects of Brownian motion, including the ballistic regime where inertia and hydrodynamics cannot be neglected, the experimental set-up and its limitations, and the development of the methodologies which allows to extract the mechanical properties of the fluids surrounding the probe.

OTI has provided results that can have an impact in fundamental aspects of biophysics, such as the influence of internal processes in the mechanical properties of biomaterials, the understanding of colloid–polymer interactions, or in the non-affine behavior of biopolymers. Moreover, the investigations of biomaterials through this set-up can contribute to potential applications in medicine and biotechnology. Through microscale engineering, scientists can influence how cells behave, move, and receive medication. By precisely designing the architecture of these materials through their internal structures, the expected biological responses can be achieved, even through energy release or enzymatic activity (Joyner et al. 2020). A typical example is the mechanical properties of fibrin, as an essential component of clots (Weisel 2004). The function of the clots depends on their viscoelastic properties, which may be associated with different pathological conditions, such as hemorrhagic risk, myocardial infarction or cancer development (Boccaccio 2006). Therefore, because high-frequency microrheology allows accessing the mechanical properties on the microscale, it reveals itself as a useful tool for bioengineering, since the mechanical testing and the developing of modeling methods are needed to understand the structures of the biomaterials, from the nano scale to the structural level (Meyers et al. 2011).

## Data Availability

No datasets were generated or analysed during the current study.
